# Multi-centre randomised controlled trial comparing arthroscopic hip surgery to physiotherapist-led care for femoroacetabular impingement (FAI) syndrome on hip cartilage metabolism: the Australian FASHIoN trial

**DOI:** 10.1186/s12891-021-04576-z

**Published:** 2021-08-16

**Authors:** David J. Hunter, Jillian Eyles, Nicholas J. Murphy, Libby Spiers, Alexander Burns, Emily Davidson, Edward Dickenson, Camdon Fary, Nadine E. Foster, Jurgen Fripp, Damian R. Griffin, Michelle Hall, Young Jo Kim, James M. Linklater, Robert Molnar, Ales Neubert, Rachel L. O’Connell, John O’Donnell, Michael O’Sullivan, Sunny Randhawa, Stephan Reichenbach, Florian Schmaranzer, Parminder Singh, Phong Tran, David Wilson, Honglin Zhang, Kim L. Bennell

**Affiliations:** 1 Institute of Bone and Joint Research, Kolling Institute of Medical Research, University of Sydney, Camperdown, Australia; 2 Department of Rheumatology, Royal North Shore Hospital, Sydney, Australia; 3 Department of Orthopaedic Surgery, Gosford and Wyong Hospitals, Gosford, New South Wales Australia; 4 Department of Physiotherapy, Centre for Health, Exercise and Sports Medicine, University of Melbourne, Parkville, Australia; 5 Orthopaedics ACT, 90 Corinna St, Canberra, 2603 Australia; 6 Department of Radiology, Royal Prince Alfred Hospital, Sydney, New South Wales 2035 Australia; 7 Warwick Medical School, University of Warwick, Coventry, UK; 8 University Hospitals of Coventry and Warwickshire NHS Trust, Coventry, UK; 9 Department of Orthopaedic Surgery, Western Health, Melbourne, Australia; 10 Australian Institute for Musculoskeletal Science (AIMSS), The University of Melbourne and Western Health, St. Albans, VIC Australia; 11 STARS Education and Research Alliance, School of Health and Rehabilitation Sciences, University of Queensland, St Lucia, Australia; 12 Primary Care Centre Versus Arthritis, School of Medicine, Keele University, Newcastle upon Tyne, UK; 13 The Australian e-Health Research Centre, CSIRO Health and Biosecurity, Brisbane, Australia; 14 Department of Orthopedic Surgery, Boston Children’s Hospital, 300 Longwood Avenue, Boston, MA 02115 USA; 15 Department of Musculoskeletal Imaging, Castlereagh Imaging, St Leonards, New South Wales Australia; 16 Sydney Orthopaedic Trauma & Reconstructive Surgery, Sydney, New South Wales Australia; 17 NHMRC Clinical Trials Centre, University of Sydney, Camperdown, Australia; 18 Hip Arthroscopy Australia, 21 Erin St, Richmond, Victoria Australia; 19 St Vincent’s Private Hospital, 159 Grey St, East Melbourne, Victoria Australia; 20 North Sydney Orthopaedic and Sports Medicine Centre, North Sydney, New South Wales Australia; 21 Macquarie University Hospital, 3 Technology Pl, Macquarie University, Macquarie Park, NSW 2109 Australia; 22 Institute of Social and Preventive Medicine, University of Bern, Bern, Switzerland; 23 Department of Rheumatology, Immunology and Allergology, University Hospital and University of Bern, Bern, Switzerland; 24 Department Diagnostic, Interventional and Paediatric Radiology, Inselspital, Bern University Hospital, University of Bern, Bern, Switzerland; 25 Maroondah Hospital, Eastern Health, Davey Drive, Ringwood East, Melbourne, Victoria 3135 Australia; 26 Department of Orthopaedics, Center for Hip Health and Mobility, University of British Columbia, Vancouver, BC Canada

**Keywords:** Arthroscopy, dGEMRIC, Femoroacetabular impingement syndrome, FAI, Hip, Physiotherapy, Surgery

## Abstract

**Background:**

Arthroscopic surgery for femoroacetabular impingement syndrome (FAI) is known to lead to self-reported symptom improvement. In the context of surgical interventions with known contextual effects and no true sham comparator trials, it is important to ascertain outcomes that are less susceptible to placebo effects. The primary aim of this trial was to determine if study participants with FAI who have hip arthroscopy demonstrate greater improvements in delayed gadolinium-enhanced magnetic resonance imaging (MRI) of cartilage (dGEMRIC) index between baseline and 12 months, compared to participants who undergo physiotherapist-led management.

**Methods:**

Multi-centre, pragmatic, two-arm superiority randomised controlled trial comparing physiotherapist-led management to hip arthroscopy for FAI. FAI participants were recruited from participating orthopaedic surgeons clinics, and randomly allocated to receive either physiotherapist-led conservative care or surgery. The surgical intervention was arthroscopic FAI surgery. The physiotherapist-led conservative management was an individualised physiotherapy program, named Personalised Hip Therapy (PHT). The primary outcome measure was change in dGEMRIC score between baseline and 12 months. Secondary outcomes included a range of patient-reported outcomes and structural measures relevant to FAI pathoanatomy and hip osteoarthritis development. Interventions were compared by intention-to-treat analysis.

**Results:**

Ninety-nine participants were recruited, of mean age 33 years and 58% male. Primary outcome data were available for 53 participants (27 in surgical group, 26 in PHT). The adjusted group difference in change at 12 months in dGEMRIC was -59 ms (95%CI − 137.9 to - 19.6) (*p* = 0.14) favouring PHT. Hip-related quality of life (iHOT-33) showed improvements in both groups with the adjusted between-group difference at 12 months showing a statistically and clinically important improvement in arthroscopy of 14 units (95% CI 5.6 to 23.9) (*p* = 0.003).

**Conclusion:**

The primary outcome of dGEMRIC showed no statistically significant difference between PHT and arthroscopic hip surgery at 12 months of follow-up. Patients treated with surgery reported greater benefits in symptoms at 12 months compared to PHT, but these benefits are not explained by better hip cartilage metabolism.

**Trial registration details:**

Australia New Zealand Clinical Trials Registry reference: ACTRN12615001177549. Trial registered 2/11/2015.

**Supplementary Information:**

The online version contains supplementary material available at 10.1186/s12891-021-04576-z.

## Background

Femoroacetabular impingement (FAI) syndrome is a motion-related clinical disorder of the hip with a triad of symptoms, clinical signs, and imaging findings [1]. It is a highly prevalent cause of hip pain in active young adults [2] and considered to be a primary contributor to the development of hip osteoarthritis (OA) [3]. It represents symptomatic premature contact between the proximal femur and acetabulum with two morphologic patterns described: cam and pincer. The repetitive abutment of the proximal femur against the acetabular rim, applying shear forces to the acetabular labrum and/or cartilage, is believed to lead to hip OA [4–6].

Both because of the symptoms associated with FAI and the purported linkages prognostically to hip OA, there is increasing interest in treating the syndrome. Currently, the most popular management approaches include surgery, mainly hip arthroscopy, and physiotherapist-led conservative care [7, 8]. Physiotherapist-led conservative care usually encompasses a multi-faceted approach, including an assessment of the patient’s pain, function and hip range of motion; an individualised and progressive exercise program; and education about the condition and its management [7]. Arthroscopic FAI surgery involves resection of the cam and/or pincer morphology, often with surgical repair of concomitant FAI-associated soft tissue pathology, such as acetabular labral tears and chondral defects. Numbers of arthroscopic hip surgeries have been increasing rapidly [8]. Several randomised controlled trials have recently been conducted comparing interventions for FAI [9–13].

The primary outcome for most of these RCTs was self-reported and possibly influenced by placebo effects. In the context of surgical interventions with known contextual effects approaching the minimum clinically important difference [14] and no true sham-comparator trials [13, 15], it is important to ascertain outcomes that are less susceptible to placebo effects. Furthermore, given the potential causative role that FAI plays in hip OA, an important criterion by which each treatment must be evaluated is its effect on the risk of future hip OA. If there are interventions that can not only reduce FAI symptoms but also prevent or slow the onset of OA, it would make a dramatic difference in the interventions recommended for those at risk [1].

A substantial problem in preventive trials in OA is that meaningful differences between treatment groups can take several decades to occur. It is in this context that this RCT measured several structural outcomes relevant to the pathogenesis of hip OA, with the aim of determining whether hip arthroscopy leads to greater improvements in hip cartilage metabolism than physiotherapist-led conservative care by 12 months follow up and hence differ in their effect on the risk of future hip OA. A prior non-randomized study [16] suggested that joint preserving surgery for FAI was associated with a decline in measures of cartilage metabolism at 1 year but the comparative effects of physiotherapy are unknown.

The primary objective was to compare change in hip cartilage mean dGEMRIC (delayed gadolinium-enhanced MRI of cartilage) score for a region of interest (ROI), including both acetabular and femoral head cartilages at the chondrolabral transitional zone between baseline and 12 months follow-up between the surgery versus conservative care groups. Several secondary objectives were pursued that are detailed in the protocol paper [17].

## Methods

The Australian FASHIoN trial was a pragmatic, assessor- and statistician-blinded, two-arm superiority RCT. The Australian FASHIoN [17] (Australian and New Zealand Clinical Trials Registration Number: ACTRN12615001177549, registered 2/11/2015) trial was conducted according to the same protocol for trial design and interventions as the parallel UK FASHIoN trial (Trial Registration number: ISRCTN64081839) [18]. Whereas the primary outcome of the UK FASHIoN trial was patient-reported hip-related quality of life (iHOT 33) at 12 months, the primary outcome for this Australian FASHIoN trial was hip cartilage metabolism at 12 months measured using MRI.

The protocol paper provides further detail on the design [17]. This trial was conducted in compliance with the Australian National Health and Medical Research Council (NHMRC) National Statement on Ethical Conduct in Human Research (2007), the Note for Guidance on Good Clinical Practice (CPMP/ICH-135/95), and the conditions of the ethics approval granted by St Vincent’s Hospital Human Research Ethics Committee (HREC/14/SVH/343). Results for this trial are reported in accordance with the CONSORT statement (see checklist in Additional file 1: Appendix 1).

### Participants and sites

Participants were recruited through public and private clinics across ten sites in Australia following a diagnosis of FAI by one of eight study orthopaedic surgeons. The personalised hip therapy (PHT) program was administered at 24 private physiotherapy clinics located throughout NSW, Victoria and ACT. Inclusion criteria were: age ≥ 16 years, hip pain, cam and/or pincer morphology on imaging (alpha angle > 55° and/or Lateral Centre Edge Angle (LCEA) > 40° or other radiographic sign of pincer morphology including positive cross-over sign), and the treating surgeon believing the patient would benefit from arthroscopic surgery. Potential participants were excluded if they had evidence of pre-existing OA (Tonnis grade > 1 [19] or < 2 mm joint space width on pelvic radiograph), previous significant hip pathology, injury, or shape-changing hip surgery.

### Randomisation, allocation and blinding

Randomisation to either arthroscopic hip surgery or physiotherapist-led conservative care occurred in a 1:1 ratio using a computer-generated minimisation sequence (adaptive stratified sampling) with study hospital site and type of FAI (cam, pincer or mixed FAI) as factors [17]. Allocation concealment was preserved by having randomisation codes held by an external biostatistician. At randomisation, participants were given a study ID that was used on all trial documentation.

Neither participants, nor treating surgeons/physiotherapists, could be blinded to treatment allocation. The treating surgeons and physiotherapists took no part in outcome assessment for the trial. Imaging analyses were performed in a blinded fashion. The patient-reported outcome data were collected via online surveys and postal questionnaires and entered onto the central trial database by a research assistant blinded to treatment allocation.

### Interventions

#### Arthroscopic hip surgery

Arthroscopic surgery was standardised and performed by one of eight orthopaedic surgeons experienced in arthroscopic hip surgery for FAI [17]. Participants could access surgery through either the public healthcare system, with no out-of-pocket cost, or through the private healthcare system, typically associated with additional out-of-pocket costs, depending on the hospital site from which they were recruited. After giving written informed consent for the procedure, patients underwent routine preoperative care, including an anaesthetic consultation to assess surgical fitness. Surgery was performed under general anaesthesia in either a lateral or supine position. Arthroscopic portals were established in the central and peripheral compartment under radiographic guidance, according to each surgeon’s usual practice. Shape abnormalities and consequent labral and cartilage pathologies were treated. Bony resection at the acetabular rim and the head-neck junction were assessed by intraoperative image intensifier radiograph and/or satisfactory impingement free range of movement of the hip. Osteo-integrative anchors were used during the surgical procedure to avoid issues with post-operative MRI quality and dGEMRIC accuracy.

Patients were discharged from hospital when they could walk safely with crutches (usually within 24 h). A protocol for post-operative rehabilitation was not specified, although patients were instructed to follow the usual post-operative rehabilitation protocol recommended by their surgeon. Physiotherapists providing post-operative rehabilitation care were distinct from those providing the physiotherapy-led conservative care in this trial to avoid contamination between groups.

To assess the fidelity of the treatment received to the prescribed surgical protocol, an international panel of 5 surgeons specialised in hip arthroscopy reviewed all surgical cases. Operation notes, intraoperative images, and postoperative MRI scans were used to evaluate the adequacy of the surgical intervention using techniques previously reported [12].

#### Physiotherapy-led conservative care

The physiotherapist-led conservative care was called Personalised Hip Therapy (PHT) [7, 17]. It was designed to represent a consensus on the best conservative care for FAI by an international panel of physiotherapists, physicians, and surgeons. The PHT program was provided at no cost to participants and delivered by experienced musculoskeletal physiotherapists in private practice who were trained in its delivery. Training involved a one-day course explaining the rationale for the FASHIoN trial and the PHT program, and instruction on how to record data related to fidelity of the intervention delivery. Participants were offered a minimum of six PHT sessions during the first 12 weeks of the trial, commencing as soon after randomisation as practicable. If needed, participants had additional PHT sessions between 12 weeks and 6 months, up to a total maximum of ten sessions. Further physiotherapy treatment beyond the ten sessions provided by the PHT physiotherapist was not part of the PHT protocol and was recorded as a co-intervention.

The PHT program encompassed a multi-faceted approach, beginning with an assessment of the patient’s pain, function and hip range of motion. The core aspects of the program included (i) an individualised and progressive exercise program supervised by a physiotherapist, (ii) education about the condition and its management, and (iii) advice regarding pain relief which could include referral to the participants’ General Practitioner, or if necessary referral for an ultrasound-guided intra-articular steroid injection to enable participants to engage in the exercise program where pain would otherwise prevent them from doing so. Physiotherapists were provided with a set of recommended exercises as part of the program, from which they prescribed and progressed appropriate exercises for each participant’s stage of rehabilitation. Participants were given a logbook to record the exercises they completed at home. Data from the logbook were not collected by researchers, but were used to enhance physiotherapist-patient communication and facilitate exercise progression and adherence to the program.

The key features of the exercise program were individualisation, progression and supervision; thus, evidence of these features was sought from individual participant PHT case report forms (CRFs). A randomly selected sample of 10 CRFs (at least one from each of the PHT physiotherapists) was assessed by two members of the panel that developed the PHT protocol [7] to assess treatment fidelity and the remaining CRFs by two Australian investigators.

### Outcomes

Participants received an MRI of their hip using standardised sequences [17] at baseline and 12 months on one of three 3 T scanners (Siemens Prisma, Siemens Skyra or Phillips Ingenia). Because of its proven reliability, the dGEMRIC technique was used for quantification and detection of changes in the glycosaminoglycan (GAG) content of the hip joint cartilage [20–23]. To this end, participants received an intravenous injection of the contrast agent; 0.2 mmol/kg bodyweight of Dotarem (Gd-DOTA; Guerbet, Cedex, France) or Magnevist (Gd-DTPA; Berlex Labs, Wayne, NJ). Following the injection of the contrast agent, participants walked for 15 min, after which MRI scanning occurred and dGEMRIC sequences were acquired 45–60 min post-injection. The primary outcome measure for analysis was the between-group difference in change from baseline to 12 months in the average T_1_ relaxation time, assessed with dGEMRIC, for a region of interest (ROI) comprising both acetabular and femoral head cartilages at the chondrolabral transitional zone of the mid-sagittal plane. The between-group difference in the change from baseline to 12-months in separate acetabular and femoral head cartilage ROIs at the chondrolabral transitional zones were analysed as a secondary outcome. In addition, the 12-month change in standardised dGEMRIC z-scores for the acetabular and femoral head cartilage ROIs at the chondrolabral junction were also assessed as secondary outcomes [24, 25].

Secondary outcomes included hip joint structural change between baseline and 12 months as demonstrated by the semi-quantitative whole hip OA MRI Score (HOAMS), a validated and reliable semi-quantitative whole hip OA MRI scoring system [26]. Participants also received standardised plain radiographs, comprising supine anteroposterior (AP) pelvis, false profile, and 45-degree modified Dunn views. Plain radiographic (measured using Hip2Norm [27]) and MRI measures of alpha angle, acetabular depth, femoral (baseline only) and acetabular version were reported at baseline and 12 months.

**Fig. 1:**
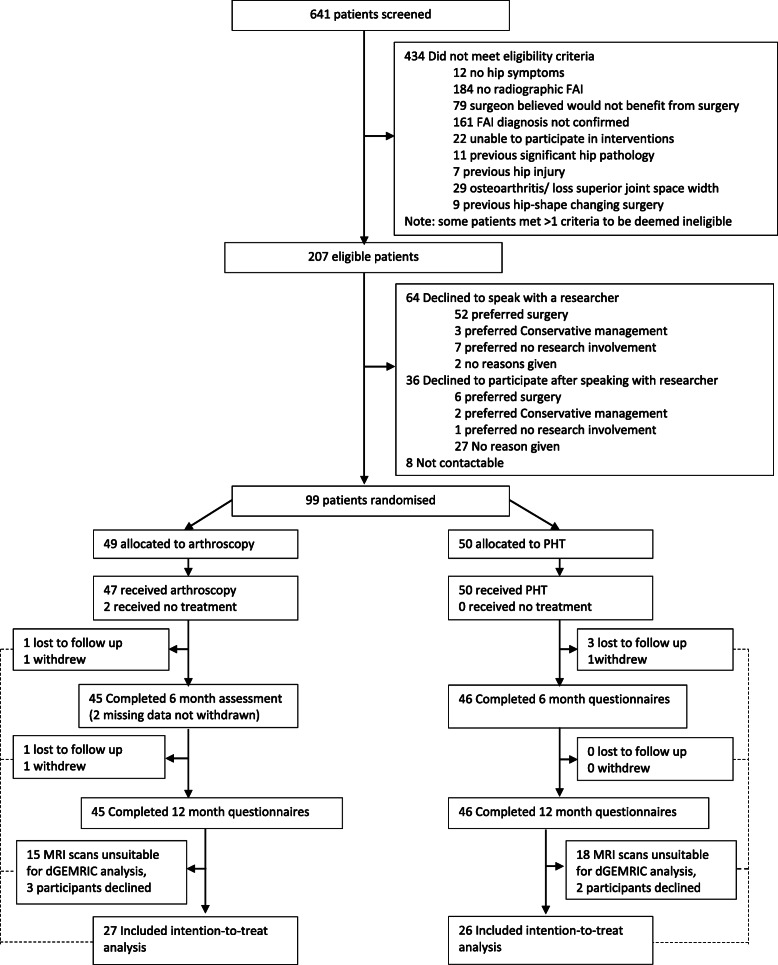
Study Flow Diagram

A range of other secondary outcomes were collected including change in: hip related quality of life as measured by the international Hip Outcome Tool-33 (iHOT-33) [28], the Hip disability and Osteoarthritis Outcome Score (HOOS) [29–31] EQ-5D [32], and the 12-Item Short Form Health Survey (SF-12) [33]; patient-perceived overall improvement following intervention measured using a Global Improvement Scale (GIS); and patient satisfaction with care and treatment results measured on a five-point Likert scale. Participants completed the Modified UCLA (University of California Los Angeles) activity score [34]. Age of symptom onset was calculated as age of presentation minus reported duration of symptoms. Procedures for measurement and analysis of each of the secondary outcomes are described in greater detail in the protocol paper [17]. The number and type of adverse events was recorded for all participants up to 12 months. For further details, see the study flow diagram (Fig. 1).

### Statistical analysis

The primary intention to treat analysis was the change in the dGEMRIC index score for a combined acetabular and femoral head cartilage ROI from baseline to 12 months, with the difference in mean change between the two intervention groups presented with a 95% confidence interval and compared using an independent samples t-test [17]. This strategy also applied for the standardised dGEMRIC z-scores. Analysis including adjustment for baseline dGEMRIC index score and relevant baseline characteristics performed using Analysis of Covariance with the change from baseline modelled as the dependent variable. This strategy was also applied for the dGEMRIC index score of the separate acetabular and femoral head cartilage ROIs and the standardized dGEMRIC z-scores.

This strategy was also used for analysis of other approximately normally distributed secondary outcome measures, including those related to plain radiography, MRI, and the patient reported outcomes (iHOT-33, HOOS, EQ-5D and SF-12). Differences in dichotomous outcome variables such as adverse events, complications related to the trial interventions and the need for further procedures were compared between groups using chi-squared tests (or Fisher’s exact test). Although our inferences are drawn from the intention-to-treat analysis, we also performed a pre-specified per-protocol analyses. We performed pre-specified exploratory subgroup analyses by FAI type and by whether referral to the trial was via the private or public healthcare systems. If participant adherence to, or completion of the PHT proved to be more problematic than expected, we intended to augment the planned analysis with a complier average causal effect (CACE) analysis.

*Economic and biomechanical analyses* will be reported in a subsequent publication.

### Sample size

Sample size calculations for the primary outcome were based on a statistical power of 90%, and two-sided significance of 5% significance level. With a sample size of 54 patients in each group, we expected to detect a difference of at least 50 ms between two intervention groups at 12 months based on a standard deviation (SD) of 80 ms for the dGEMRIC index [22, 35]. 50 ms was chosen as clinically significant based upon an increased risk of subsequent total hip replacement [36]. We aimed to recruit a total of 140 participants to allow for a drop-out rate of 20%, including 5% cross-over to the surgical group. (See Additional file 1: Appendix 3 for details on reduced sample size, which came about in large part as a consequence of changes in Medicare funding for hp. arthroscopy shortly after the study commenced).

## Results

Ninety-nine participants (of whom we have primary outcome data on 53-see Flow Chart (Fig. 1)) were recruited from February 17, 2015 to December 30th 2017, of which 49 were allocated to arthroscopy, 50 allocated to PHT and 3 patients crossed over from PHT to arthroscopy during the 12 months of the study. Our participants had a mean age of 33 years, were predominantly male (58%), 20% had bilateral symptoms, the median duration of symptoms was 20 months, and cam type FAI was predominant (63%). More details on the participants are included in Table 1. The average time to commencement of treatment after randomisation was 12.5 (SD (8.1) weeks for arthroscopy and 4.7 (SD 2.3) weeks for PHT. Those with complete dGEMRIC data were broadly similar to those that had missing dGEMRIC data (Supplementary Table 8). The between group change in dGEMRIC is outlined in Table 2. The adjusted group difference in the primary dGEMRIC combined outcomes was -59 ms (95%CI − 137.9 to - 19.6) (*p* = 0.14), the direction of effect favouring PHT in terms of hip cartilage metabolism but was not statistically significant. Between group effects for acetabular and femoral ROI were similar, with more acetabular change and again non-significant. A per-protocol analysis (*n* = 39) found similar results (Supplementary Table 1).

**Table 1:** Baseline Characteristics (*N* = 99)

*Characteristic*		*Arthroscopy* *N = 49*	*PHT* *N = 50*	*Total* *N = 99*
Age (years, mean [SD])		32.9 (11.8)	32.9 (9.1)	32.9 (10.5)
Gender:	Male	31 (63%)	26 (52%)	57 (58%)
	Female	18 (37%)	24 (48%)	42 (42%)
Current smoker:	Yes	5 (10%)	5 (10%)	10 (10%)
	No	41 (84%)	44 (88%)	85 (86%)
	Missing	3 (6%)	1 (2%)	4 (4%)
Hip side to be considered for treatment:	right	22 (45%)	24 (48%)	46 (46%)
	left	27 (55%)	26 (52%)	53 (54%)
Bilateral symptoms:	Yes	9 (18%)	11 (22%)	20 (20%)
	No	40 (82%)	39 (78%)	79 (80%)
Duration of hip symptoms (mths, median [min, max])		24.0 (2.0, 84.0)	18.0 (2.5, 120.0)	20.0 (2.0, 120.0)
Type of FAI:	Pincer	9 (18%)	9 (18%)	18 (18%)
	Mixed	10 (20%)	9 (18%)	19 (19%)
	Cam	30 (61%)	32 (64%)	62 (63%)
Units of alcohol in average week (median [min, max])		3.0 (0.0, 16.0)	2.0 (0.0, 12.0)	2.0 (0.0, 16.0)
Diabetic:	Yes	1 (2%)		1 (1%)
	No	46 (94%)	49 (98%)	95 (96%)
	Missing	2 (4%)	1 (2%)	3 (3%)
Diagnosed chronic renal failure:	No	47 (96%)	49 (98%)	96 (97%)
	Missing	2 (4%)	1 (2%)	3 (3%)
Physical activity (UCLA score, mean [SD])		7.5 (2.6)	7.2 (2.8)	7.3 (2.7)
HOOS pain (mean [SD])		59.4 (18.4)	57.4 (18.9)	58.4 (18.6)
HOOS Symptom (mean [SD])		49.7 (17.5)	50.7 (20.9)	50.2 (19.2)
HOOS ADL (mean [SD])		69.2 (18.0)	65.9 (20.4)	67.5 (19.3)
HOOS Sport & Recreation (mean [SD])		47.9 (22.4)	46.9 (23.6)	47.4 (22.9)
HOOS Quality of Life (mean [SD])		33.6 (16.1)	30.1 (17.6)	31.8 (16.9)
Hip related Quality of Life (i-HOT-33, (mean [SD]))		43.5 (17.6)	40.4 (20.0)	41.9 (18.8)
SF-12 PCS (mean [SD])		40 (7.8)	39 (8.4)	39 (8.1)
SF-12 MCS (mean [SD])		49 (11.2)	48 (10.8)	49 (10.9)
EQ-5D-5L index score (mean [SD])		0.575 (0.21)	0.544 (0.23)	0.559 (0.22)
EQ-5D-5L VAS (mean [SD])		68.9 (16.3)	68.8 (14.2)	68.8 (15.2)
Maximum MRI alpha angle (mean [SD])		70.2 (11.9)	70.6 (15.6)	70.4 (13.9)
dGEMRIC Combined (mean [SD])		679.6 (118.6)	667.0 (127.4)	673.6 (121.8)
dGEMRIC Acetabular ROI (mean [SD])		661.2 (121.1)	637.2 (145.3)	649.7 (132.4)
dGEMRIC Femoral ROI (mean [SD])		698.9 (137.0)	698.4 (125.4)	698.7 (130.2)
dGEMRIC Z-score (mean [SD])		−0.49 (0.70)	−0.45 (0.67)	− 0.47 (0.68)
Total AP anterior coverage (%, mean [SD])		27.2 (8.2)	24.7 (6.2)	25.9 (7.3)
Total AP posterior coverage (%, mean [SD])		47.4 (8.1)	45.0 (8.3)	46.2 (8.3)
Total Femur coverage (%, mean [SD])		82.4 (7.6)	81.0 (7.3)	81.7 (7.4)
LCE (%, mean [SD])		37.1 (5.4)	34.7 (6.6)	35.8 (6.2)
LCE	< 25		2 (4%)	2 (2%)
	25+	47 (96%)	48 (96%)	95 (96%)
	Missing	2 (4%)		2 (2%)
Radiographic Measurements				
Acetabular Index (%, mean [SD])		2.5 (4.2)	4.7 (4.8)	3.6 (4.6)
ACM angle (%, mean [SD])		45.0 (2.5)	44.7 (3.6)	44.8 (3.1)
Extrusion Index (%, mean [SD])		15.0 (4.5)	17.0 (5.7)	16.0 (5.3)
Cross-over-sign	Yes	31 (63%)	38 (76%)	69 (70%)
	No	16 (33%)	12 (24%)	28 (28%)
	Missing	2 (4%)		2 (2%)
Retroversion Index (%, median [min, max])		6.7 (0.0, 46.0)	11.1 (0.0, 54.7)	10.2 (0.0, 54.7)
Posterior wall sign	Yes	29 (59%)	37 (74%)	66 (67%)
	No	18 (37%)	13 (26%)	31 (31%)
	Missing	2 (4%)		2 (2%)

Data are number (%) unless otherwise indicated. *UCLA* University of California Los Angeles. *iHOT-33* International Hip Outcome Tool. *SF-12* 12-item Short Form Health Survey. *PCS* Physical component score. *MCS* Mental component score

*LCEA* Lateral centre edge angle. Hip Osteoarthritis MRI Scoring System (HOAMS). ROI = region of interest

*ACM* Angle constructed by the following points: (A) lateral edge of the acetabulum, (M) midpoint of a line connecting the lateral and the inferior acetabular edge, (C) point of the bony acetabulum intersecting the perpendicular line relative to line AM through point M

Hip2Norm results are for study hip

**Table 2:** Primary outcome of hip cartilage metabolism assessed by dGEMRIC (ms): Change from Baseline (B) to 12 month (12 M) assessments (*N* = 53)

*Outcome*	*Arthroscopy (N = 27)*		*PHT (N = 26)*		*Arthroscopy - PHT*			
	*n*	*Mean (SD or 95% CI)*	*n*	*Mean (SD or 95% CI)*	*Unadjusted difference*	*P-value*^a^	*Adjusted difference (95% CI)*	*P-value*^b^
**dGEMRIC Combined (primary outcome)**								
Baseline	26	679.6 (118.6)	24	667.0 (127.4)				
12 month	21	677.0 (122.8)	23	722.8 (145.7)				
Change: 12 M-B	20	−14.8 (−73.8–44.2)	21	48.4 (−16.6–113.5)	−63.2	0.142	−59.1 (− 137.9–19.6)	0.137
**dGEMRIC Acetabular ROI**								
Baseline	26	661.2 (121.1)	24	637.2 (145.3)				
12 month	21	618.5 (130.5)	23	651.8 (145.0)				
Change: 12 M-B	20	−63.0 (−120.2 - -5.9)	21	7.1 (−55.5–69.7)	−70.1	0.093	−58.9 (− 134.8–17.1)	0.125
**dGEMRIC Femoral ROI**								
Baseline	26	698.9 (137.0)	24	698.4 (125.4)				
12 month	21	735.8 (135.6)	23	789.8 (170.8)				
Change: 12 M-B	20	34.1 (−37.6–105.7)	21	84.2 (8.9–159.5)	−50.1	0.321	−55.4 (− 149.5–38.6)	0.240
**dGEMRIC Z-score**								
Baseline	26	−0.49 (0.70)	24	−0.45 (0.67)				
12 month	21	− 0.50 (0.51)	23	−0.69 (0.54)				
Change: 12 M-B	20	0.16 (−0.21–0.53)	21	−0.20 (− 0.63–0.23)	0.36	0.199	0.24 (−0.11–0.58)	0.169

^a^Paired t-test

^b^Based on regression model including adjustment for baseline

A range of secondary outcomes are presented in Table 3. iHOT-33 showed improvements in both groups with the adjusted group difference showing a statistically and clinically important greater improvement in the arthroscopy group of 14.2 units (95% CI 5.6 to 23.9) (*p* = 0.003). There were similarly greater improvements in quality of life (EQ-5D-5L), HOOS pain and symptoms in the arthroscopy group compared to the PHT group. There were statistically significant between group differences in the maximum MRI alpha angle (*p* = 0.001), along with radiographic between group differences in LCEA and extrusion index favouring arthroscopy.

**Table 3:** Secondary outcomes: Change from Baseline (B) to 6 month (6 M) and 12 month (12 M) assessments (*N* = 99

*Outcome*	*Arthroscopy* *N = 49*		*PHT* *N = 50*		*Arthroscopy - PHT*			
	*n*	*Mean (SD or 95% CI)*	*n*	*Mean (SD or 95% CI)*	*Unadjusted difference*	*P-value*^*a*^	*Adjusted difference (95% CI)*	*P-value*^*c*^
**iHOT-33**								
Baseline	48	43.5 (17.6)	50	40.4 (20.0)				
6 month	44	62.2 (21.2)	42	57.5 (24.9)				
Change: 6 M-B	44	18.6 (13.0–24.2)	42	13.3 (7.2–19.3)	5.4	0.194	5.2 (−2.7–13.2)	0.196
12 month	45	72.9 (21.8)	46	56.8 (28.8)				
Change: 12 M-B	45	29.6 (22.9–36.3)	46	15.4 (8.8–22.1)	14.2	0.003	14.7 (5.6–23.9)	0.002
**EQ-5D-5L index score**								
Baseline	48	0.575 (0.206)	50	0.544 (0.231)				
6 month	44	0.703 (0.177)	41	0.682 (0.155)				
Change: 6 M-B	44	0.121 (0.068–0.174)	41	0.089 (0.033–0.145)	0.032	0.405	0.026 (−0.035–0.087)	0.398
12 month	45	0.770 (0.156)	46	0.653 (0.249)				
Change: 12 M-B	45	0.194 (0.131–0.256)	46	0.101 (0.033–0.169)	0.093	0.046	0.106 (0.029–0.183)	0.007
**EQ5D-VAS**								
Baseline	48	68.9 (16.3)	50	68.8 (14.2)				
6 month	45	72.7 (16.8)	42	71.7 (16.1)				
Change: 6 M-B	45	3.7 (−1.5–9.0)	42	1.3 (−4.4–7.0)	2.4	0.530	1.6 (−4.9–8.1)	0.627
12 month	45	76.9 (15.2)	46	74.7 (14.6)				
Change: 12 M-B	45	7.9 (2.4–13.3)	46	5.8 (1.0–10.6)	2.1	0.570	2.1 (−3.7–7.9)	0.470
**HOOS Pain**								
Baseline	47	59.4 (18.4)	50	57.4 (18.9)				
6 month	45	74.0 (17.8)	45	67.6 (23.5)				
Change: 6 M-B	43	13.2 (7.0–19.4)	45	10.9 (5.7–16.1)	2.3	0.567	3.7 (−3.8–11.2)	0.327
12 month	41	83.9 (16.0)	42	71.3 (19.9)				
Change: 12 M-B	41	24.8 (17.7–32.0)	42	11.1 (5.2–17.1)	13.7	0.004	12.9 (5.4–20.5)	0.001
**HOOS Symptom**								
Baseline	47	49.7 (17.5)	50	50.7 (20.9)				
6 month	45	64.4 (19.7)	45	59.8 (25.6)				
Change: 6 M-B	43	14.4 (8.3–20.5)	45	8.7 (3.5–13.8)	5.8	0.149	5.3 (−2.3–12.9)	0.168
12 month	41	73.8 (18.8)	42	63.8 (22.8)				
Change: 12 M-B	41	23.2 (16.1–30.3)	42	9.6 (3.4–15.8)	13.5	0.005	11.7 (3.3–20.1)	0.007
**HOOS ADL**								
Baseline	47	69.2 (18.0)	50	65.9 (20.4)				
6 month	45	80.8 (18.2)	45	75.0 (24.0)				
Change: 6 M-B	43	11.1 (5.1–17.2)	45	9.2 (4.7–13.6)	2.0	0.591	3.1 (−3.9–10.1)	0.377
12 month	41	89.7 (14.3)	42	76.6 (21.4)				
Change: 12 M-B	41	20.4 (14.6–26.2)	42	7.4 (2.4–12.3)	13.0	0.001	13.0 (6.4–19.7)	0.000
**HOOS Sport and Recreation**								
Baseline	47	47.9 (22.4)	50	46.9 (23.6)				
6 month	45	61.1 (19.9)	45	58.5 (27.9)				
Change: 6 M-B	43	12.8 (6.0–19.6)	45	12.5 (5.9–19.1)	0.3	0.951	1.1 (−7.4–9.6)	0.803
12 month	41	75.2 (17.5)	42	62.5 (25.4)				
Change: 12 M-B	41	28.4 (20.7–36.0)	42	13.2 (5.9–20.6)	15.1	0.005	13.7 (4.9–22.4)	0.003
**HOOS Quality of Life**								
Baseline	47	33.6 (16.1)	50	30.1 (17.6)				
6 month	45	47.2 (24.2)	45	42.2 (23.3)				
Change: 6 M-B	43	13.4 (6.5–20.2)	45	13.3 (8.4–18.3)	0.0	0.993	0.8 (−7.4–9.1)	0.845
12 month	41	62.7 (25.3)	42	46.0 (24.3)				
Change: 12 M-B	41	29.3 (21.7–36.8)	42	15.6 (8.4–22.9)	13.6	0.010	14.9 (4.9–24.8)	0.004
**MAX MRI alpha angle**								
Baseline	47	70.2 (11.9)	49	70.6 (15.6)				
12 month	43	62.7 (16.9)	41	69.2 (16.2)				0.039^b^
Change: 12 M-B	43	−7.5 (−11.4 - -3.6)	40	−0.1 (−1.2–1.0)	−7.4	0.001	−7.4 (−11.6 - -3.3)	0.001
**Radiographic (Hip2Norm) Measures**								
**Total AP anterior coverage**								
Baseline	47	27.2 (8.2)	50	24.7 (6.2)				
12 month	42	26.7 (5.9)	43	25.2 (6.0)				
Change: 12 M-B	42	−0.2 (−2.1–1.7)	43	0.9 (−1.4–3.2)	− 1.1	0.452	0.4 (−1.9–2.7)	0.706
**Total AP posterior coverage**								
Baseline	47	47.4 (8.1)	50	45.0 (8.3)				
12 month	42	47.0 (8.5)	43	44.6 (8.8)				
Change: 12 M-B	42	−0.7 (−1.8–0.5)	43	−0.4 (−2.6–1.8)	−0.3	0.820	0.2 (−2.2–2.6)	0.845
**Total Femur coverage**								
Baseline	47	82.4 (7.6)	50	81.0 (7.3)				
12 month	42	79.8 (6.7)	43	81.0 (6.7)				
Change: 12 M-B	42	−2.4 (−5.0–0.2)	43	0.3 (−1.6–2.2)	−2.7	0.097	−1.8 (−4.4–0.7)	0.153
**LCE**								
Baseline	47	37.1 (5.4)	50	34.7 (6.6)				
12 month	42	34.2 (6.2)	43	34.0 (5.6)				
Change: 12 M-B	42	−2.9 (−4.3 - -1.5)	43	−0.4 (−1.4–0.7)	−2.5	0.005	−1.8 (−3.5 - − 0.2)	0.031
**Acetabular Index**								
Baseline	47	2.5 (4.2)	50	4.7 (4.8)				
12 month	42	4.0 (5.0)	43	5.4 (4.4)				
Change: 12 M-B	42	1.2 (0.1–2.4)	43	1.0 (−0.2–2.1)	0.3	0.728	-0.2 (−1.7–1.3)	0.790
**ACM angle**								
Baseline	47	45.0 (2.5)	50	44.7 (3.6)				
12 month	42	44.6 (2.8)	43	44.7 (3.1)				
Change: 12 M-B	42	−0.3 (−1.1–0.5)	43	0.0 (−0.8–0.9)	−0.3	0.598	−0.2 (− 1.2–0.8)	0.688
**Extrusion Index**								
Baseline	47	15.0 (4.5)	50	17.0 (5.7)				
12 month	42	18.2 (5.2)	43	17.4 (4.9)				
Change: 12 M-B	42	3.3 (1.9–4.7)	43	0.1 (−0.8–1.1)	3.2	0.000	2.5 (0.9–4.0)	0.002
**Retroversion Index**								
Baseline	47	11.3 (12.8)	50	14.7 (14.4)				
12 month	42	9.3 (10.7)	43	11.3 (14.3)				
Change: 12 M-B	42	−0.8 (−3.9–2.2)	43	−2.5 (−6.7–1.7)	1.6	0.527	0.0 (− 4.6–4.6)	0.997

*iHOT-33* International Hip; Hip Osteoarthritis MRI Scoring System (HOAMS)

^a^Paired t-test

^b^Wilcoxon rank-sum test

^c^Based on regression model including adjustment for baseline

Results of the semi-quantitative MRI analysis (HOAMS) are presented in Table 4. These demonstrate worsening in cartilage score (*p* = 0.002), an increase in number of regions worsened for cartilage score (*p* = 0.0002), and an increase in the number of worsened regions for labral score (*p* = 0.0009) for arthroscopy compared to PHT. In contrast, there was a reduction in the number of regions affected by osteophytes (*p* = 0.01) for hip arthroscopy compared to PHT. No participants had loose bodies, attrition or dysplasia. Baseline HOAMS scores are included in Supplementary Table 9.

**Table 4:** HOAMS (semi-quantitative MRI) outcomes: Changes over 12 months. Values are number (%), unless otherwise indicated

MRI feature/category	Arthroscopy *N* = 42	PHT *N* = 43	Difference, % (95% CI)	*P*-value
Cartilage				
Change across all regions (excluding within-grade change):				
No change	29 (69%)	43 (100%)		0.0002^a^
Worsening in 1 subregion	11 (26%)	0		
Worsening in 2 subregions	2 (5%)	0		
Any worsening	13 (31)	0	31 (15–47)	
Change across all regions (including within-grade change):				
Improvement in 1 subregion+ worsening in 1 subregion	1 (2%)	0		
No change	26 (62%)	41 (95%)		
Worsening in 1 subregion only	11 (26%)	2 (5%)		
Worsening in 2 subregions only	3 (7%)	0		
Worsening in 3+ subregions only	1 (2%)	0		
Any worsening	16 (38%)	2 (5%)	33 (15–52)	0.0002^b^
BML				
Change in no. of subregions affected by any lesion:				
Improvement	1 (2%)	1 (2%)		0.61^a^
No change	38 (91%)	40 (93%)		
Worsening in 1 subregion	1 (2%)	2 (5%)		
Worsening in 2 subregions	2 (5%)	0		
Maximum change in BML score across all subregions:				
No change^c^	37 (88%)	40 (93%)		0.66^a^
Within-grade worsening	2 (5%)	0		
Worsening by 1 grade	3 (7%)	3 (7%)		
Any subregion with improvement (including within-grade changes) in BML	2 (5%)	4 (9%)	−4 (−18–9)	
Any subregion with worsening (including within-grade changes) in BML	5 (12%)	3 (7%)	5 (−10–20)	
Subchondral cyst				
Change in no. of subregions affected:				
Improvement	5 (12%)	0		0.11^a^
No change	33 (78%)	39 (91%)		
Worsening in 1 subregion	4 (10%)	3 (7%)		
Worsening in 2 subregions	0	0		
Worsening in 3 subregions	0	1 (2%)		
Maximum change in score across all subregions:				
No change^c^	38 (90%)	37 (86%)		0.75^a^
Within-grade worsening	0	2 (5%)		
Worsening by 1 grade	4 (10%)	4 (9%)		
Any subregion with improvement (including within-grade changes)	7 (17%)	3 (7%)	10 (−6–26)	
Any subregion with worsening (including within-grade changes)	4 (10%)	6 (14%)	−4 (−20–12)	
Osteophyte				
Increase in no. locations affected by any osteophyte:	0	0		
Change in no. of locations affected by any osteophyte:				
Improvement 1 subregion	6 (14%)	0		0.01^b^
No Change	36 (86%)	42 (100%)		
Maximum change in osteophyte score ≥ 1 across all subregions:	0	0		
Labrum				
Maximum change in score across all subregions:				
No change^c^	17 (40%)	34 (81%)		0.0009^a^
Within-grade worsening	12 (29%)	3 (7%)		
Worsening by 1 grade	11 (26%)	5 (12%)		
Worsening by 2 grades	2 (5%)	0		
Any subregion with improvement (including within-grade changes)	7 (17%)	2 (5%)	12 (−3–27)	
Any subregion with worsening (including within-grade changes)	25 (60%)	8 (19%)	40 (19–62)	
Synovitis				
Maximum change in score across all subregions:				
No change^c^	14 (33%)	23 (58%)		0.08^a^
Within-grade worsening	7 (17%)	5 (12%)		
Worsening by 1 grade	19 (45%)	11 (28%)		
Worsening by 2 grades	2 (5%)	1 (2%)		
Any subregion with improvement (including within-grade changes)	10 (24%)	10 (24%)	−0 (−21–20)	
Any subregion with worsening (including within-grade changes)	28 (67%)	17 (41%)	25 (2–48)	

^a^Ordinal chi-squared test

^b^Chi-squared test

^c^Includes within-grade improvement

Per protocol dGEMRIC analysis is broadly consistent with the results presented in Table 2 (Supplementary Table 1). Subgroup analyses comparing FAI type (cam vs mixed vs pincer) (Supplemental Table 2), age (Supplementary Table 3) and public vs private study hospital sites (Supplementary Table 5) did not demonstrate any significant between group differences. Participants with a baseline dGEMRIC index above the median (indicating better cartilage metabolism) demonstrated a significant between group difference in change of − 110.7 ms, favouring PHT (*p* = 0.035) (Supplementary Table 4).

Surgery for the majority of surgical participants demonstrated satisfactory fidelity (84%) but was deemed inadequate for seven participants (16%) (Supplementary Table 6). PHT was also judged as satisfactory in terms of intervention fidelity for the majority of participants (82% of PHT participants).

PHT was generally well tolerated with 25 participants (53%) complaining of muscle soreness from exercise. In the hip arthroscopy surgery group, 33% of participants complained of numbness in the groin, leg or foot; and 31% had some problems as a consequence of taking pain medication (Supplementary Table 7).

## Discussion

The primary outcome of hip cartilage metabolism dGEMRIC showed no statistically significant difference between PHT and arthroscopic hip surgery at 12 months follow-up. Since the loss of GAG in cartilage is an early OA-related change [37], dGEMRIC enables the likelihood of future OA development to be compared between interventions after a relatively short period [35]. It is important to be cognisant that a range of issues led to a reduction in the required sample size and there is a trend in cartilage metabolism favouring PHT. The baseline standard deviation in our trial participants (~ 120 ms) is greater than the 80 ms used in our sample size calculation, suggesting heterogeneity of this measure in our trial population which would have further underpowered our ability to detect a significant difference and raising the distinct possibility that the lack of statistical significance observed is a Type 2 error. The mean difference in the arthroscopic treatment group was small and the between group difference was comparable with the estimate that we used for the sample size calculation. The results of semi-quantitative MRI analyses demonstrated worse cartilage and labral scores in the arthroscopic group at 12 months.

Some potential reasons for the findings relate to MRI cartilage metabolism. During hip arthroscopy, surgeons often perform an acetabular chondroplasty / microfracture / or chondral repair. Furthermore, during a labral repair the procedure involves separating any chondro-labral adhesions or scar with a rasp prior to repair. All of these procedures could adversely affect labral and cartilage scores. Another potential mechanism is that surgery introduces a transient inflammatory state that may adversely affect cartilage biochemical content in the short to medium term. The decline that we have seen in dGEMRIC indices has been found in prior investigations and the magnitude of the decline found in our surgical group is broadly consistent with prior studies [16]. Further, there may be positive effects of exercise on glycosaminoglycan content consistent with what we have found in our PHT group [38, 39].

Several randomised controlled trials have recently been conducted comparing interventions for FAI [9–13]. Potentially as a consequence of their differing designs, including a variety of comparator interventions, study settings, and eligibility criteria; the RCTs to date show both surgery and conservative care can lead to average benefits overall, but have had inconsistent conclusions regarding superiority of one versus another. In our trial, from a patient–reported symptom standpoint, the range of secondary outcomes demonstrated statistically and clinically important improvements with significant between group differences favouring surgery [14] in IHOT-33, quality of life, HOOS pain and symptoms at 12 months, similar to the UK FASHIoN trial [12]. Intriguingly, while trending in the same direction, there were no significant differences in improvements between groups at 6 months. Both groups demonstrated symptom improvements at 12 months, but the arthroscopic group consistently demonstrated greater benefit. The magnitude of effect and separation from PHT comparator is broadly consistent with prior randomised controlled trials [12, 40]. The results of semi-quantitative MRI analyses demonstrated worse cartilage and labral scores in the arthroscopic group at 12 months. In addition, participants with a baseline dGEMRIC above the median, indicating better cartilage metabolism, also demonstrated a clear separation in between group dGEMRIC difference favouring the PHT arm. The magnitude of that difference (111 ms) is what would be considered clinically meaningful, as a threshold of 50 ms has prognostic value for an increased risk of subsequent total hip replacement in hip dysplasia [36]. Several studies on hip dysplasia and FAI have shown that dGEMRIC as a metric can detect cartilage changes in early OA, and predict the outcomes of joint preserving surgery, as well as the likelihood of surgical failure and requirement for total hip replacement [35, 36, 41, 42]. In contrast, semi-quantitative MRI scores for osteophytes improved in the surgical arm, but this may reflect their resection during the arthroscopic procedure.

In this regard, further assessment of the longer term clinical outcomes is critical. This will be forthcoming for these trial participants and in addition analyses focused on identifying whether there is any association between structural changes found on the MRI at 12 months and the longer term (3–5 years) self-reported symptom outcome measures will be critical in understanding the prognostic value of these cartilage metabolism findings.

There are a number of strengths of our trial that warrant mentioning. The design was rigorous and the outcomes are reliable [12, 17]. The demographics of our trial population are broadly consistent with prior studies, in particular the UK FASHIoN trial [12, 40]. In addition, there are a number of limitations. Unfortunately, we were not able to recruit the targeted sample size in large part because of a change in funding for hip arthroscopy by the Australian government that impacted our recruitment. In addition, a number of concerns around gadolinium based contrast agents and potential deposition in the brain [43] led to a change from Magnevist to Dotarem. In addition, the currently reported followup duration is short at only 12 months.

The need for RCTs to determine the most effective approach for the management of FAI is well-recognised [44]. While this trial suggests short-term (12 months) changes in cartilage metabolism, the longer term prognostic significance of these as it relates to clinical outcomes remains unknown. The data should be available within the next 2 to 3 years. Furthermore, the biomechanical effects of surgery and their prognostic value also will be determined as will health economic analyses. Further research, ideally with a credible sham surgery comparator is needed to identify whether the superiority of surgery at 12 months in these RCTs is explained by non-specific effects [45].

## Conclusions

In conclusion, the primary outcome of dGEMRIC showed no statistically significant difference between arthroscopic hip surgery and conservative care (PHT) at 12 months of follow-up, although this may represent a type 2 error. Patients treated with surgery reported greater improvements in symptoms at 12 months compared to PHT. Both interventions provide benefits, on average, for patients but surgery more so than PHT. This trial adds new information that shows the patient reported benefits of surgery are not explained by nor linked to better hip cartilage metabolism at 12 months.

## Supplementary Information

**Additional file 1: Appendix 1**. CONSORT Checklist. **Appendix 2**. MRI missing data. **Appendix 3**. Details on rationale for reduced sample size. **Appendix 4**. Statistical analysis plan. **Appendix 5**. Protocol.

**Additional file 2: Supplementary Table 1.** FASHION Per-protocol analysis: dGEMRIC (ms): Change from Baseline (B) and 12 month (12M) assessments (*N*=39). **Supplementary Table 2.** dGEMRIC (ms) Combined: Change from Baseline (B) and 12 month (12M) assessments (*N*=53). Subgroup analysis FAI type. **Supplementary Table 3.** FASHION: dGEMRIC (ms) Combined: Change from Baseline (B) and 12 month (12M) assessments (*N*=53). **Supplementary Table 4.** FASHION: dGEMRIC (ms) Combined: Change from Baseline (B) and 12 month (12M) assessments (*N*=50). **Supplementary Table 5.** dGEMRIC Combined (ms): Change from Baseline (B) and 12 month (12M) assessments (*N*=53). Subgroup analysis: Public vs Private. **Supplementary Table 6.** Intervention Fidelity. **Supplementary Table 7.** All patient-reported adverse events n (%). **Supplementary Table 8.** FASHION: Comparison of Baseline Characteristics for patients with dGEMRIC data vs missing (*N*=99). **Supplementary Table 9.** Baseline MRI HOAMS features. Numbers are n (%).

## Data Availability

**Data are available upon reasonable request** Deidentified participant data are available from the corresponding author for collaboration. Additional information including the protocol [17] and statistical analysis plan is also available.
