# Supplementary material for: Multi-centre randomised controlled trial comparing arthroscopic hip surgery to physiotherapist-led care for femoroacetabular impingement (FAI) syndrome on hip cartilage metabolism: the Australian FASHIoN trial
Source: BMC Musculoskelet Disord. 2021 Aug 16;22:697. doi: 10.1186/s12891-021-04576-z (PMC8369620; doi:10.1186/s12891-021-04576-z)
Supplement: Supplementary file 1 — Additional file 1: Appendix 1. CONSORT Checklist. Appendix 2. MRI missing data. Appendix 3. Details on rationale for reduced sample size. Appendix 4. Statistical analysis plan. Appendix 5. Protocol. [file 12891_2021_4576_MOESM1_ESM.docx]

**Appendix Contents**

Table of Contents

[Appendix 1. CONSORT Checklist 2](#_Toc46823477)

[Appendix 2. MRI missing data 3](#_Toc46823478)

[Appendix 3. Details on rationale for reduced sample size 4](#_Toc46823479)

[Appendix 4. Statistical analysis plan 4](#_Toc46823480)

[Appendix 5. Protocol 4](#_Toc46823481)

# Appendix 1. CONSORT Checklist

# Appendix 2. MRI missing data

The first 39 participants in this study were scanned using Magnevist (Gd-DTPA; Berlex Labs, Wayne, NJ) at baseline, however received a different gadolinium based contrast agent (GBCA), Dotarem (Gd-DOTA; Guerbet, Cedex, France) at their 12-month scan. This occurred due to changing radiological practices associated with concern over the unknown clinical significance of linear GBCA deposition in the brain (1). Additionally, MRI technician error resulted in these participants being administered a half dose (0.1 mmol/kg bodyweight) of GBCA. The dGEMRIC results from these participants had to be excluded given the systematic error introduced by a change in contrast agent between baseline and 12-month scans and use of an unvalidated GBCA dosing protocol.

A further five participants scanned on the Canberra Phillips scanner were excluded because the MRI technician decided to alter the TR between dGEMRIC series to reduce scan time.

# Appendix 3. Details on the rationale for reduced sample size

Recruitment for the trial began in February 17, 2015. Shortly after recruitment began, the Australian government announced it would cut funding for hip arthroscopy in the management of FAI. In outlining the 2016-2017 budget, the Medicare benefit schedule states in relation to hip arthroscopy:

Revised MBS items for hip arthroscopy services which will limit the items to the management of non-femoroacetabular impingement indications.

These changes took effect as of 1 November, 2016.

Despite protests, this MBS change stayed in effect and markedly impacted our ability to recruit participants for this trial, particularly through private healthcare settings. As a consequence, we became increasingly reliant on the public system, but despite our best efforts, we were not able to get to the pre-planned sample size required before the study budget ran out.

# Appendix 4. Statistical analysis plan

# Appendix 5. Protocol

1. Olchowy C, Cebulski K, Lasecki M, Chaber R, Olchowy A, Kalwak K, et al. The presence of the gadolinium-based contrast agent depositions in the brain and symptoms of gadolinium neurotoxicity - A systematic review. PloS one. 2017;12(2):e0171704.
