# Supplementary material for: Multi-centre randomised controlled trial comparing arthroscopic hip surgery to physiotherapist-led care for femoroacetabular impingement (FAI) syndrome on hip cartilage metabolism: the Australian FASHIoN trial
Source: BMC Musculoskelet Disord. 2021 Aug 16;22:697. doi: 10.1186/s12891-021-04576-z (PMC8369620; doi:10.1186/s12891-021-04576-z)
Supplement: Supplementary file 2 — Additional file 2: Supplementary Table 1. FASHION Per-protocol analysis: dGEMRIC (ms): Change from Baseline (B) and 12 month (12M) assessments (N=39). Supplementary Table 2. dGEMRIC (ms) Combined: Change from Baseline (B) and 12 month (12M) assessments (N=53). Subgroup analysis FAI type. Supplementary Table 3. FASHION: dGEMRIC (ms) Combined: Change from Baseline (B) and 12 month (12M) assessments (N=53). Supplementary Table 4. FASHION: dGEMRIC (ms) Combined: Change from Baseline (B) and 12 month (12M) assessments (N=50). Supplementary Table 5. dGEMRIC Combined (ms): Change from Baseline (B) and 12 month (12M) assessments (N=53). Subgroup analysis: Public vs Private. Supplementary Table 6. Intervention Fidelity. Supplementary Table 7. All patient-reported adverse events n (%). Supplementary Table 8. FASHION: Comparison of Baseline Characteristics for patients with dGEMRIC data vs missing (N=99). Supplementary Table 9. Baseline MRI HOAMS features. Numbers are n (%). [file 12891_2021_4576_MOESM2_ESM.docx]

| \| ***Supplementary Table 1. FASHION Per-protocol analysis: dGEMRIC (ms): Change from Baseline (B) and 12 month (12M) assessments (N=39)*** \| \| --- \|  \| *Outcome* \| *Arthroscopy (N = 20)* \| \| *PHT (N = 19)* \| \| *Unadjusted Diff* \| \| *Adjusted Diff* \| \| \| --- \| --- \| --- \| --- \| --- \| --- \| --- \| --- \| --- \| \|  \| *n* \| *Mean (SD or 95% CI)* \| *n* \| *Mean (SD or 95% CI)* \| *Mean* \| *P-value** \| *Difference (95% CI)* \| *P-value§* \| \| **dGEMRIC Combined** \|  \|  \|  \|  \|  \|  \|  \|  \| \| Baseline \| 19 \| 705.3 (113.4) \| 17 \| 701.5 (110.8) \|  \|  \|  \|  \| \| 12 month \| 18 \| 704.0 (101.0) \| 17 \| 723.6 (106.4) \|  \|  \|  \|  \| \| Change: 12M-B \| 17 \| -7.8 (-77.1 - 61.5) \| 15 \| 29.4 (-44.1 - 102.9) \| -37.2 \| 0.438 \| -30.7 (-106.2 - 44.8) \| 0.413 \| \| **dGEMRIC Acetabular ROI** \|  \|  \|  \|  \|  \|  \|  \|  \| \| Baseline \| 19 \| 693.2 (107.3) \| 17 \| 666.3 (131.2) \|  \|  \|  \|  \| \| 12 month \| 18 \| 647.0 (114.3) \| 17 \| 647.0 (114.6) \|  \|  \|  \|  \| \| Change: 12M-B \| 17 \| -56.8 (-123.0 - 9.4) \| 15 \| -11.1 (-85.0 - 62.9) \| -45.7 \| 0.332 \| -23.5 (-102.1 - 55.0) \| 0.544 \| \| **dGEMRIC Femoral ROI** \|  \|  \|  \|  \|  \|  \|  \|  \| \| Baseline \| 19 \| 717.6 (141.8) \| 17 \| 738.6 (100.7) \|  \|  \|  \|  \| \| 12 month \| 18 \| 762.0 (111.5) \| 17 \| 794.3 (121.5) \|  \|  \|  \|  \| \| Change: 12M-B \| 17 \| 42.4 (-41.6 - 126.3) \| 15 \| 60.9 (-21.6 - 143.5) \| -18.6 \| 0.741 \| -37.2 (-125.1 - 50.7) \| 0.394 \| \| **dGEMRIC Z-score** \|  \|  \|  \|  \|  \|  \|  \|  \| \| Baseline \| 19 \| -0.47 (0.80) \| 17 \| -0.33 (0.74) \|  \|  \|  \|  \| \| 12 month \| 18 \| -0.52 (0.41) \| 17 \| -0.69 (0.56) \|  \|  \|  \|  \| \| Change: 12M-B \| 17 \| 0.13 (-0.25 - 0.51) \| 15 \| -0.32 (-0.88 - 0.25) \| 0.44 \| 0.164 \| 0.23 (-0.14 - 0.60) \| 0.209 \|  \| ****Paired t-test*** \| \| --- \| \| ***§Based on regression model including adjustment for baseline*** \|   ***Supplementary Table 2. dGEMRIC (ms) Combined: Change from Baseline (B) and 12 month (12M) assessments (N=53). Subgroup analysis FAI type*** | | | | | | | | | | |
| --- | --- | --- | --- | --- | --- | --- | --- | --- | --- | --- | --- | --- | --- | --- | --- | --- | --- | --- | --- | --- | --- | --- | --- | --- | --- | --- | --- | --- | --- | --- | --- | --- | --- | --- | --- | --- | --- | --- | --- | --- | --- | --- | --- | --- | --- | --- | --- | --- | --- | --- | --- | --- | --- | --- | --- | --- | --- | --- | --- | --- | --- | --- | --- | --- | --- | --- | --- | --- | --- | --- | --- | --- | --- | --- | --- | --- | --- | --- | --- | --- | --- | --- | --- | --- | --- | --- | --- | --- | --- | --- | --- | --- | --- | --- | --- | --- | --- | --- | --- | --- | --- | --- | --- | --- | --- | --- | --- | --- | --- | --- | --- | --- | --- | --- | --- | --- | --- | --- | --- | --- | --- | --- | --- | --- | --- | --- | --- | --- | --- | --- | --- | --- | --- | --- | --- | --- | --- | --- | --- | --- | --- | --- | --- | --- | --- | --- | --- | --- | --- | --- | --- | --- | --- | --- | --- | --- | --- | --- | --- | --- | --- | --- | --- | --- | --- | --- | --- | --- | --- | --- | --- | --- | --- | --- | --- |
|  | | | | | | | | | | |
| *FAI type* | *Arthroscopy (N = 27)* | | *PHT (N = 26)* | | *Unadjusted Diff* | | *Adjusted Diff* | | *Interaction* |  |
|  | *n* | *Mean (SD or 95% CI)* | *n* | *Mean (SD or 95% CI)* | *Mean* | *P-value** | *Difference (95% CI)* | *P-value§* | *P-value§* |  |
| **Cam** |  |  |  |  |  |  |  |  | 0.691 |  |
| Baseline | 17 | 672.6 (126.1) | 16 | 700.1 (114.0) |  |  |  |  |  |  |
| 12 month | 13 | 679.1 (146.2) | 15 | 720.9 (133.8) |  |  |  |  |  |  |
| Change: 12M-B | 12 | -1.8 (-79.6 - 76.1) | 14 | 26.2 (-50.3 - 102.7) | -28.0 | 0.584 | -37.9 (-136.1 - 60.4) | 0.433 |  |  |
| **Mixed** |  |  |  |  |  |  |  |  |  |  |
| Baseline | 6 | 684.5 (135.2) | 2 | 698.9 (126.1) |  |  |  |  |  |  |
| 12 month | 5 | 683.3 (96.6) | 3 | 773.1 (100.9) |  |  |  |  |  |  |
| Change: 12M-B | 5 | -23.1 (-237.7 - 191.4) | 2 | 116.3 (-1904.8 - 2137.4) | -139.4 | 0.408 | -130.7 (-376.3 - 115.0) | 0.214 |  |  |
| **Pincer** |  |  |  |  |  |  |  |  |  |  |
| Baseline | 3 | 709.7 (33.2) | 6 | 568.4 (130.3) |  |  |  |  |  |  |
| 12 month | 3 | 656.9 (56.9) | 5 | 698.2 (215.2) |  |  |  |  |  |  |
| Change: 12M-B | 3 | -52.7 (-235.7 - 130.2) | 5 | 83.6 (-119.7 - 286.8) | -136.3 | 0.232 | -241.5 (-573.4 - 90.4) | 0.120 |  |  |
| ****Paired t-test*** | | | | | | | | | | |
| ***§Based on regression model including adjustment for baseline*** | | | | | | | | | | |

| ***Supplementary Table 3. FASHION: dGEMRIC (ms) Combined: Change from Baseline (B) and 12 month (12M) assessments (N=53).*** | | | | | | | | | | |
| --- | --- | --- | --- | --- | --- | --- | --- | --- | --- | --- |
| ***Subgroup analysis: Age*** | | | | | | | | | | |
| *Age:* | *Arthroscopy (N = 27)* | | *PHT (N = 26)* | | *Unadjusted Diff* | | *Adjusted Diff* | | *Interaction* |  |
|  | *n* | *Mean (SD or 95% CI)* | *n* | *Mean (SD or 95% CI)* | *Mean* | *P-value** | *Difference (95% CI)* | *P-value§* | *P-value§* |  |
| **<=32 yrs** |  |  |  |  |  |  |  |  | 0.565 |  |
| Baseline | 15 | 695.7 (138.8) | 10 | 690.5 (106.5) |  |  |  |  |  |  |
| 12 month | 10 | 642.9 (138.4) | 12 | 712.9 (124.0) |  |  |  |  |  |  |
| Change: 12M-B | 10 | -66.0 (-131.2 - -0.8) | 10 | 22.9 (-86.0 - 131.8) | -88.9 | 0.131 | -81.3 (-191.3 - 28.6) | 0.137 |  |  |
| **>32 yrs** |  |  |  |  |  |  |  |  |  |  |
| Baseline | 11 | 657.8 (85.3) | 14 | 650.3 (142.0) |  |  |  |  |  |  |
| 12 month | 11 | 707.9 (103.5) | 11 | 733.6 (171.8) |  |  |  |  |  |  |
| Change: 12M-B | 10 | 36.5 (-63.3 - 136.2) | 11 | 71.6 (-20.3 - 163.6) | -35.2 | 0.567 | -36.3 (-158.6 - 86.0) | 0.541 |  |  |
| ****Paired t-test*** | | | | | | | | | | |
| ***§Based on regression model including adjustment for baseline*** | | | | | | | | | | |

| ***Supplementary Table 4. FASHION: dGEMRIC (ms) Combined: Change from Baseline (B) and 12 month (12M) assessments (N=50)*** | | | | | | | | | | |
| --- | --- | --- | --- | --- | --- | --- | --- | --- | --- | --- |
| ***Subgroup analysis: Baseline dGEMRIC*** | | | | | | | | | | |
| *dGEMRIC:* | *Arthroscopy (N = 27)* | | *PHT (N = 26)* | | *Unadjusted Diff* | | *Adjusted Diff* | | *Interaction* |  |
|  | *n* | *Mean (SD or 95% CI)* | *n* | *Mean (SD or 95% CI)* | *Mean* | *P-value** | *Difference (95% CI)* | *P-value§* | *P-value§* |  |
| **<= Median** |  |  |  |  |  |  |  |  | 0.218 |  |
| Baseline | 13 | 588.3 (81.5) | 12 | 563.4 (82.5) |  |  |  |  |  |  |
| 12 month | 8 | 633.0 (141.9) | 11 | 649.9 (145.6) |  |  |  |  |  |  |
| Change: 12M-B | 8 | 69.5 (-31.6 - 170.6) | 11 | 66.0 (-26.5 - 158.4) | 3.6 | 0.954 | 1.4 (-133.1 - 135.8) | 0.983 |  |  |
| **> Median** |  |  |  |  |  |  |  |  |  |  |
| Baseline | 13 | 771.0 (67.7) | 12 | 770.7 (60.9) |  |  |  |  |  |  |
| 12 month | 12 | 693.7 (104.0) | 10 | 805.4 (118.8) |  |  |  |  |  |  |
| Change: 12M-B | 12 | -71.0 (-133.0 - -8.9) | 10 | 29.1 (-80.6 - 138.9) | -100.1 | 0.078 | -110.7 (-213.0 - -8.4) | 0.035 |  |  |
| ****Paired t-test*** | | | | | | | | | | |
| ***§Based on regression model including adjustment for baseline*** | | | | | | | | | | |

| ***Supplementary Table 5. dGEMRIC Combined (ms): Change from Baseline (B) and 12 month (12M) assessments (N=53). Subgroup analysis: Public vs Private*** |
| --- |
|  |

| *Hospital type:* | *Arthroscopy (N = 27)* | | *PHT (N = 26)* | | *Unadjusted Diff* | | *Adjusted Diff* | | *Interaction* |  |
| --- | --- | --- | --- | --- | --- | --- | --- | --- | --- | --- |
|  | *n* | *Mean (SD or 95% CI)* | *n* | *Mean (SD or 95% CI)* | *Mean* | *P-value** | *Difference (95% CI)* | *P-value§* | *P-value§* |  |
| **Public** |  |  |  |  |  |  |  |  | 0.873 |  |
| Baseline | 18 | 691.0 (126.4) | 19 | 666.8 (131.3) |  |  |  |  |  |  |
| 12 month | 15 | 665.7 (135.2) | 19 | 718.0 (156.5) |  |  |  |  |  |  |
| Change: 12M-B | 14 | -36.5 (-100.2 - 27.2) | 17 | 36.0 (-41.1 - 113.0) | -72.4 | 0.144 | -70.0 (-166.0 - 26.0) | 0.147 |  |  |
| **Private** |  |  |  |  |  |  |  |  |  |  |
| Baseline | 8 | 654.0 (101.6) | 5 | 667.8 (125.7) |  |  |  |  |  |  |
| 12 month | 6 | 705.2 (88.7) | 4 | 745.4 (88.7) |  |  |  |  |  |  |
| Change: 12M-B | 6 | 35.9 (-128.0 - 199.8) | 4 | 101.4 (-71.8 - 274.7) | -65.5 | 0.490 | -42.5 (-187.0 - 101.9) | 0.509 |  |  |
| ****Paired t-test*** | | | | | | | | | | |
| ***§Based on regression model including adjustment for baseline*** | | | | | | | | | | |

**Supplementary Table 6. Intervention Fidelity**

| ***Fidelity for surgical patients*** |
| --- |

|  | | *n (%)* |
| --- | --- | --- |
| Fidelity Rating | Missing | 6 |
|  | Satisfactory | 34 (72.3%) |
|  | Inadequate | 7 (14.9%) |
|  | - head not spherical | 1 |
|  | - no bony resection | 5 |
|  | - degenerative | 1 |
|  | ***Fidelity for PHT patients*** |  |
| Fidelity rating | Missing | 3 |
|  | Satisfactory | 41 (82%) |
|  | Did not have 6 contacts with physio | 3 |
|  | Treatment period >6months | 2 |
|  | Non-protocol treatment (ultrasound) | 1 |

|  |
| --- |

**Supplementary Table 7. All patient-reported adverse events n (%)**

| Patient reported adverse events | **Arthroscopy**  **n= 45** | **PHT**  **n= 47** |
| --- | --- | --- |
| Problems with pain medications | 14 (31) | 3 (6) |
| Details | 5 Gastric irritation with NSAIDs;  1 sleep disturbance from opioids;  2 constipation with opioids  5 nausea &/or vomiting with opioids;  1 drowsiness with opioids. | 2 drowsiness from Lyrica;  1 sweating, nausea, abdominal cramps, anxiety morphine. |
| Problems with hip joint injections |  | 3 (6) |
| Details |  | 1 pain injection site;  1 muscle cramps;  1 ill, hot & sweaty for 48hrs post injection. |
| Muscle soreness from exercises | 22 (49) | 25 (53) |
| Details | 22 intermittent muscle pain and delayed onset muscle soreness. | 25 delayed onset muscle soreness- resolved itself. |
| A regional pain syndrome | 0 | 1 (2) |
| Details |  | 1 Neuropathic groin pain. |
| Deep Venous Thrombosis | 0 | 0 |
|  |  |  |
| Any other complications | 7 (16) | 11 (23) |
| Details | 1 lump from cannula insertion for 1 month;  1 delayed post-op physio;  1 quads and knee pain 2-3 weeks post-op;  1 deep pain in hip after slept in prone position;  1 GP stitched small section of wound because oozing;  1 ankle felt unstable after surgical traction;  1 given inappropriate exercise program post-op- cause pain & anxiety. | 1 endoscopic inguinal ligament repair (unsure if related)*;  4 increased pain in hip after physio;  2 flare up ‘old’ knee injury;  1 back pain;  1 migraines;  1 plantar fasciitis on recommencing running. |
| Numbness in the groin leg or foot | 15 (33) |  |
| Details | 12 short term numbness in foot&/or groin that resolved in up to 4 weeks;  2 numbness over incision;  1 tingling in toes after sleeping in a strange position. |  |
| Wound infection | 3 (7) |  |
| Details | 3 superficial, treated with antibiotics, didn’t require hospitalisation or further surgery. |  |
| Hip fracture break | 0 |  |
|  |  |  |
| Further surgery | 1 (2) |  |
| Details | 1 Pain over TFL & bursa several weeks following arthroscopy- had surgical removal of bursa (unsure if related)* |  |
| Other complications unrelated to intervention | 7 (15) | 4 (9) |
| Details | 1 Inguinal hernia repair;  1 thyrotoxicosis;  1 fall with foot fracture;  1 rotator cuff reconstruction;  1 fall on operated shoulder (same participant as above);  1 C5/6/7 fusion;  1 surgery to remove testicular mass (benign). | 1 disc herniation;  2 fall with injured knees/hips- no fractures;  1 fall with ankle fracture. |
| Complications related to MRI contrast |  | 2 (4) |
| Details |  | 1 nausea & dizziness;  1 pain/tenderness arm and pectoral area, peaking around 5 days after injection. |

** Serious adverse event.one SAE

| ***Supplementary Table 8. FASHION: Comparison of Baseline Characteristics for patients with dGEMRIC data vs missing (N=99)*** |
| --- |

| *Characteristic* |  | *Missing dGEMRIC N = 46* | *Yes has dGEMRIC N = 53* | *Total N = 99* | *P-value* |
| --- | --- | --- | --- | --- | --- |
| Age (years, mean [SD]) |  | 33.9 (9.6) | 32.0 (11.1) | 32.9 (10.5) | 0.366 |
| Gender: | Male | 23 (50%) | 34 (64%) | 57 (58%) | 0.155 |
|  | Female | 23 (50%) | 19 (36%) | 42 (42%) |  |
| Curent smoker: | Yes | 4 (9%) | 6 (11%) | 10 (10%) | 0.475 |
|  | No | 39 (85%) | 46 (87%) | 85 (86%) |  |
|  | Missing | 3 (7%) | 1 (2%) | 4 (4%) |  |
| Hip side to be considered for treatment: | right | 21 (46%) | 25 (47%) | 46 (46%) | 0.880 |
|  | left | 25 (54%) | 28 (53%) | 53 (54%) |  |
| Bilateral symptoms: | Yes | 6 (13%) | 14 (26%) | 20 (20%) | 0.098 |
|  | No | 40 (87%) | 39 (74%) | 79 (80%) |  |
| Duration of hip symptoms (mths, median [min, max]) |  | 23.0 (2.0, 120.0) | 18.0 (2.5, 120.0) | 20.0 (2.0, 120.0) | 0.689 |
| Type of FAI: | Pincer | 9 (20%) | 9 (17%) | 18 (18%) | 0.743 |
|  | Mixed | 10 (22%) | 9 (17%) | 19 (19%) |  |
|  | Cam | 27 (59%) | 35 (66%) | 62 (63%) |  |
| Units of alcohol in average week (median [min, max]) |  | 3.0 (0.0, 16.0) | 2.0 (0.0, 15.0) | 2.0 (0.0, 16.0) | 0.149 |
| Diabetic: | Yes | 1 (2%) |  | 1 (1%) | 0.428 |
|  | No | 43 (93%) | 52 (98%) | 95 (96%) |  |
|  | Missing | 2 (4%) | 1 (2%) | 3 (3%) |  |
| Diagnosed chronic renal failure: | No | 44 (96%) | 52 (98%) | 96 (97%) | 0.476 |
|  | Missing | 2 (4%) | 1 (2%) | 3 (3%) |  |
| Physical activity (UCLA score, mean [SD]) |  | 7.0 (2.7) | 7.6 (2.6) | 7.3 (2.7) | 0.339 |
| HOOS pain (mean [SD]) |  | 59.4 (19.7) | 57.5 (17.8) | 58.4 (18.6) | 0.623 |
| HOOs Symptom (mean [SD]) |  | 53.4 (20.2) | 47.5 (18.1) | 50.2 (19.2) | 0.136 |
| HOOs ADL (mean [SD]) |  | 70.0 (19.8) | 65.5 (18.7) | 67.5 (19.3) | 0.254 |
| HOOs Sport & Recreation (mean [SD]) |  | 51.7 (25.0) | 43.8 (20.5) | 47.4 (22.9) | 0.088 |
| HOOs Quality of Life (mean [SD]) |  | 33.2 (17.8) | 30.7 (16.2) | 31.8 (16.9) | 0.458 |
| Hip related Quality of Life (i-HOT-33, (mean [SD])) |  | 45.5 (19.4) | 38.9 (17.9) | 41.9 (18.8) | 0.085 |
| SF-12 PCS (mean [SD]) |  | 40 (7.9) | 39 (8.3) | 39 (8.1) | 0.724 |
| SF-12 MCS (mean [SD]) |  | 50 (9.7) | 48 (11.9) | 49 (10.9) | 0.436 |
| EQ-5D-5L index score (mean [SD]) |  | 0.589 (0.21) | 0.534 (0.22) | 0.559 (0.22) | 0.219 |
| EQ-5D-5L VAS (mean [SD]) |  | 69.2 (17.0) | 68.5 (13.7) | 68.8 (15.2) | 0.806 |
| MAX MRI alpha angle (mean [SD]) |  | 71.0 (15.4) | 69.9 (12.6) | 70.4 (13.9) | 0.695 |
| **Hip2Norm** |  |  |  |  |  |
| Total AP anterior coverage (%, mean [SD]) |  | 25.4 (6.9) | 26.3 (7.7) | 25.9 (7.3) | 0.535 |
| Total AP posterior coverage (%, mean [SD]) |  | 47.3 (7.8) | 45.3 (8.5) | 46.2 (8.3) | 0.235 |
| Total Femur coverage (%, mean [SD]) |  | 82.5 (7.7) | 81.0 (7.2) | 81.7 (7.4) | 0.329 |
| LCE (%, mean [SD]) |  | 36.1 (7.1) | 35.6 (5.3) | 35.8 (6.2) | 0.742 |
| LCE | <25 | 2 (4%) |  | 2 (2%) | 0.091 |
|  | 25+ | 42 (91%) | 53 (100%) | 95 (96%) |  |
|  | Missing | 2 (4%) |  | 2 (2%) |  |
| Acetabular Index (%, mean [SD]) |  | 4.3 (5.0) | 3.1 (4.3) | 3.6 (4.6) | 0.226 |
| ACM angle (%, mean [SD]) |  | 45.0 (3.4) | 44.8 (2.8) | 44.8 (3.1) | 0.740 |
| Extrusion Index (%, mean [SD]) |  | 15.7 (6.1) | 16.3 (4.5) | 16.0 (5.3) | 0.559 |
| Cross-over-sign | Yes | 30 (65%) | 39 (74%) | 69 (70%) | 0.260 |
|  | No | 14 (30%) | 14 (26%) | 28 (28%) |  |
|  | Missing | 2 (4%) |  | 2 (2%) |  |
| Retroversion Index (%, median [min, max]) |  | 8.4 (0.0, 46.0) | 11.0 (0.0, 54.7) | 10.2 (0.0, 54.7) | 0.511 |
| Posterior wall sign | Yes | 28 (61%) | 38 (72%) | 66 (67%) | 0.216 |
|  | No | 16 (35%) | 15 (28%) | 31 (31%) |  |
|  | Missing | 2 (4%) |  | 2 (2%) |  |

| ***Data are number (%) unless otherwise indicated. UCLA=University of California Los Angeles. iHOT-33=International Hip*** |
| --- |
| ***Outcome Tool. SF-12=12-item Short Form Health Survey. PCS=physical component score. MCS=mental component score.*** |
| ***LCEA=lateral centre edge angle. Hip Osteoarthritis MRI Scoring System (HOAMS).*** |
| ***Hip2Norm results are for study hip.*** |

***Supplementary Table 9.* Baseline MRI HOAMS features. Numbers are n (%).**

| MRI feature/category | Arthroscopy  N = 47 | PHT  N = 50 |
| --- | --- | --- |
| Cartilage: |  |  |
| Maximum score: |  |  |
| 0 | 29 (62%) | 31 (62%) |
| 1 | 4 (9%) | 5 (10%) |
| 2 | 13 (28%) | 11 (22%) |
| 3 | 0 (0%) | 2 (4%) |
| 4 | 1 (2%) | 1 (2%) |
| Number of subregions with score >0 |  |  |
| 0 | 29 (62%) | 31 (62%) |
| 1-2 | 15 (32%) | 14 (28%) |
| 3+ | 3 (6%) | 5 (10%) |
| BML: |  |  |
| Maximum score: |  |  |
| 0 | 42 (89%) | 44 (88%) |
| 1 | 4 (9%) | 3 (6%) |
| 2 | 1 (2%) | 3 (6%) |
| Number of subregions affected: |  |  |
| 0 | 42 (89%) | 44 (88%) |
| 1 | 1 (2%) | 4 (8%) |
| 2 | 4 (9%) | 0 |
| 3 | 0 | 0 |
| 4 | 0 | 0 |
| 5+ | 0 | 2 (4%) |
| Subchondral cyst: |  |  |
| Maximum score: |  |  |
| 0 | 32 (68%) | 34 (68%) |
| 1 | 15 (32%) | 11 (22%) |
| 2 | 0 (0%) | 5 (10%) |
| Number of subregions with affected: |  |  |
| 0 | 32 (68%) | 34 (68%) |
| 1 | 12 (26%) | 10 (20%) |
| 2 | 3 (6%) | 4 (8%) |
| 3 | 0 | 2 (4%) |
| Osteophyte: |  |  |
| Maximum score: |  |  |
| 0 | 39 (83%) | 43 (86%) |
| 1 | 4 (9%) | 1 (2%) |
| 2 | 4 (9%) | 4 (8%) |
| 3 | 0 | 1 (2%) |
| 4 | 0 | 1 (2%) |
| Number of subregions affected: |  |  |
| 0 | 39 (83%) | 43 (86%) |
| 1 | 5 (11%) | 3 (6%) |
| 2 | 3 (6%) | 3 (6%) |
| 3+ | 0 | 1 (2%) |
| Labram |  |  |
| Maximum score: |  |  |
| 0 | 3 (6%) | 2 (4%) |
| 1 | 2 (4%) | 4 (8%) |
| 2 | 38 (81%) | 38 (76%) |
| 3 | 4 (9%) | 6 (12%) |
| Number of subregions affected: |  |  |
| 0 | 3 (6%) | 2 (4%) |
| 1 | 7 (15%) | 10 (20%) |
| 2 | 21 (45%) | 27 (54%) |
| 3 | 14 (30%) | 9 (18%) |
| 4 | 2 (4%) | 2 (4%) |
| Synovitis: |  |  |
| Maximum score: |  |  |
| 0 | 8 (17%) | 18 (37%) |
| 1 | 34 (72%) | 26 (53%) |
| 2 | 5 (11%) | 5 (10%) |
| Number of subregions affected: |  |  |
| 0 | 8 (17%) | 18 (37%) |
| 1 | 7 (15%) | 16 (33%) |
| 2 | 12 (26%) | 5 (10%) |
| 3 | 12 (26%) | 6 (12%) |
| 4 | 8 (17%) | 4 (8%) |
